# Supplementary material for: TSPAN15 interacts with BTRC to promote oesophageal squamous cell carcinoma metastasis via activating NF-κB signaling
Source: Nat Commun. 2018 Apr 12;9:1423. doi: 10.1038/s41467-018-03716-9 (PMC5897412; doi:10.1038/s41467-018-03716-9)
Supplement: Supplementary file 1 — Supplementary Information(PDF 910 kb) [file 41467_2018_3716_MOESM1_ESM.pdf]

TSPAN15 Interacts with BTRC to Promote Oesophageal Squamous Cell Carcinoma Metastasis via Activating NF-κB signaling

Zhang et al.

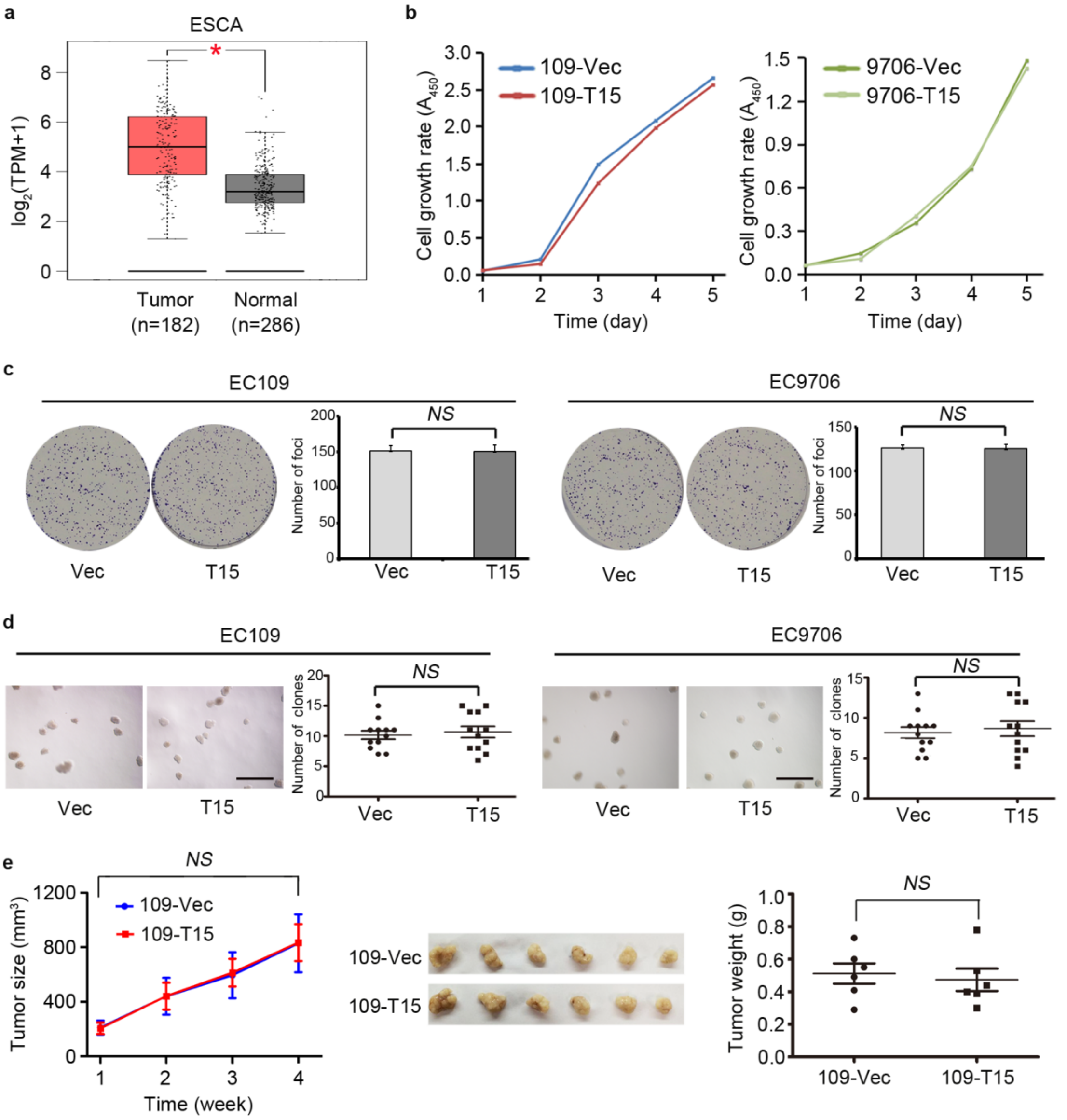

Supplementary Fig. 1. Overexpression of TSPAN15 does not affect OSCC cells proliferation. (a) RNA sequencing expression of *TSPAN15* was analyzed by GEPIA based on TCGA and GTEx data. TPM, transcripts per million. *, $P < 0.01$, one-way ANOVA. **(b)** Growth curves of *TSPAN15*-expressing cells were compared with control cells by XTT assay. Points, mean of at least three independent experiments; bars, s.d. Representatives and summary of foci formation **(c)** and colony formation in soft agar **(d)** induced by *TSPAN15*- and vector-transfected cells. Scale bar, 500 μm. The values are represented as the mean ± s.d. of three independent experiments. NS, $P > 0.05$, Student's *t*-test. **(e)** Tumor growth curves of 109-*T15* cells in nude mice were compared with 109-Vec cells by tumor xenograft experiments. Points, mean of six mice; bars, s.d. NS, $P > 0.05$, Student's *t*-test. Representatives of tumors in nude mice induced by 109-Vec or 109-*T15* cells. Tumor weights were summarized in dot chart. Bars, s.d. NS, $P > 0.05$, Student's *t*-test.

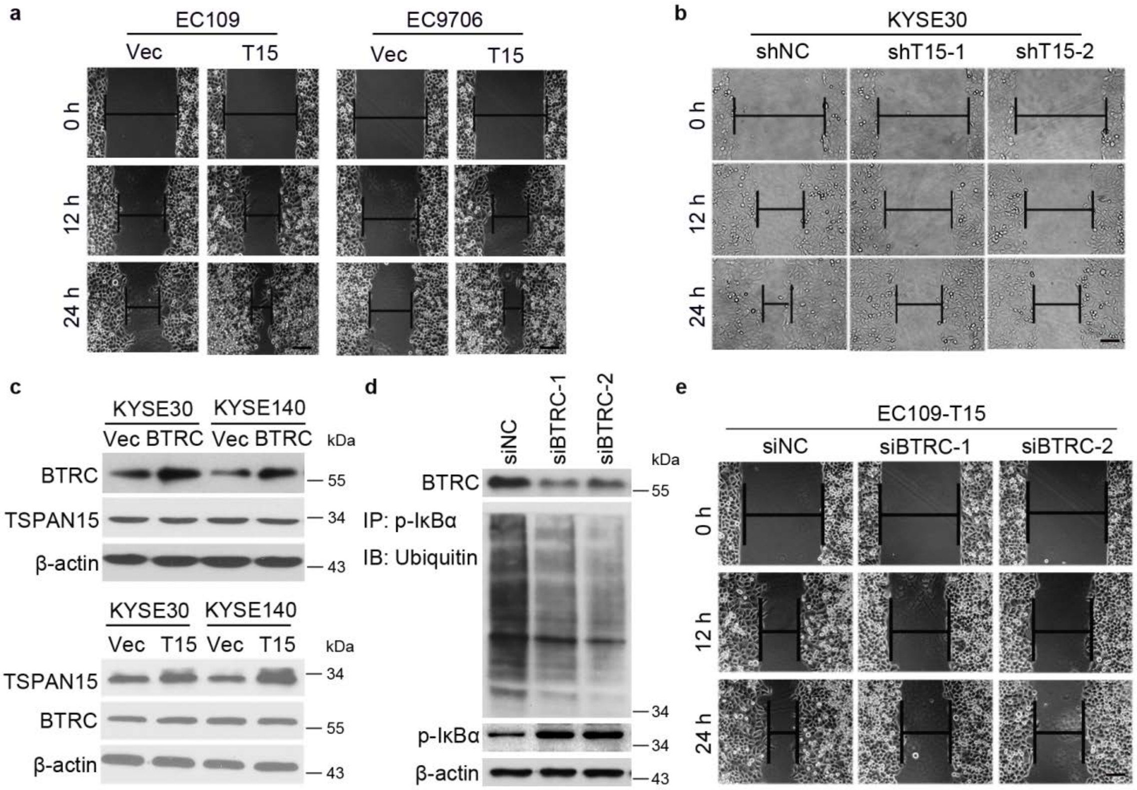

Supplementary Fig. 2. TSPAN15 promotes the migratory ability of OSCC cells via interaction with BTRC. (a) Wound-healing assay displayed that overexpression of *TSPAN15* promoted cell migration. Representative images were taken at 0 h, 12 h and 24 h after scratching. Scale bar, 200 µm. **(b)** Knockdown of *TSPAN15* effectively inhibited cell migration. Representative images were taken at 0 h, 12 h and 24 h after scratching. Scale bar, 200 µm. **(c)** Western blot analysis examined the protein level of BTRC and TSPAN15 after introduction of *BTRC* (*upper*), and the protein expression of TSPAN15 and BTRC after transfection of *TSPAN15* (*lower*). **(d)** MG-132-induced accumulation of polyubiquitinated p-IκBα was impaired by knockdown of *BTRC* in KYSE30 cells. β-actin was used as a loading control. **(e)** Knockdown of *BTRC* in 109-*T15* inhibited cell migration compared to control group. Representative images were taken at 0 h, 12 h and 24 h after scratching. Scale bar, 200 µm.

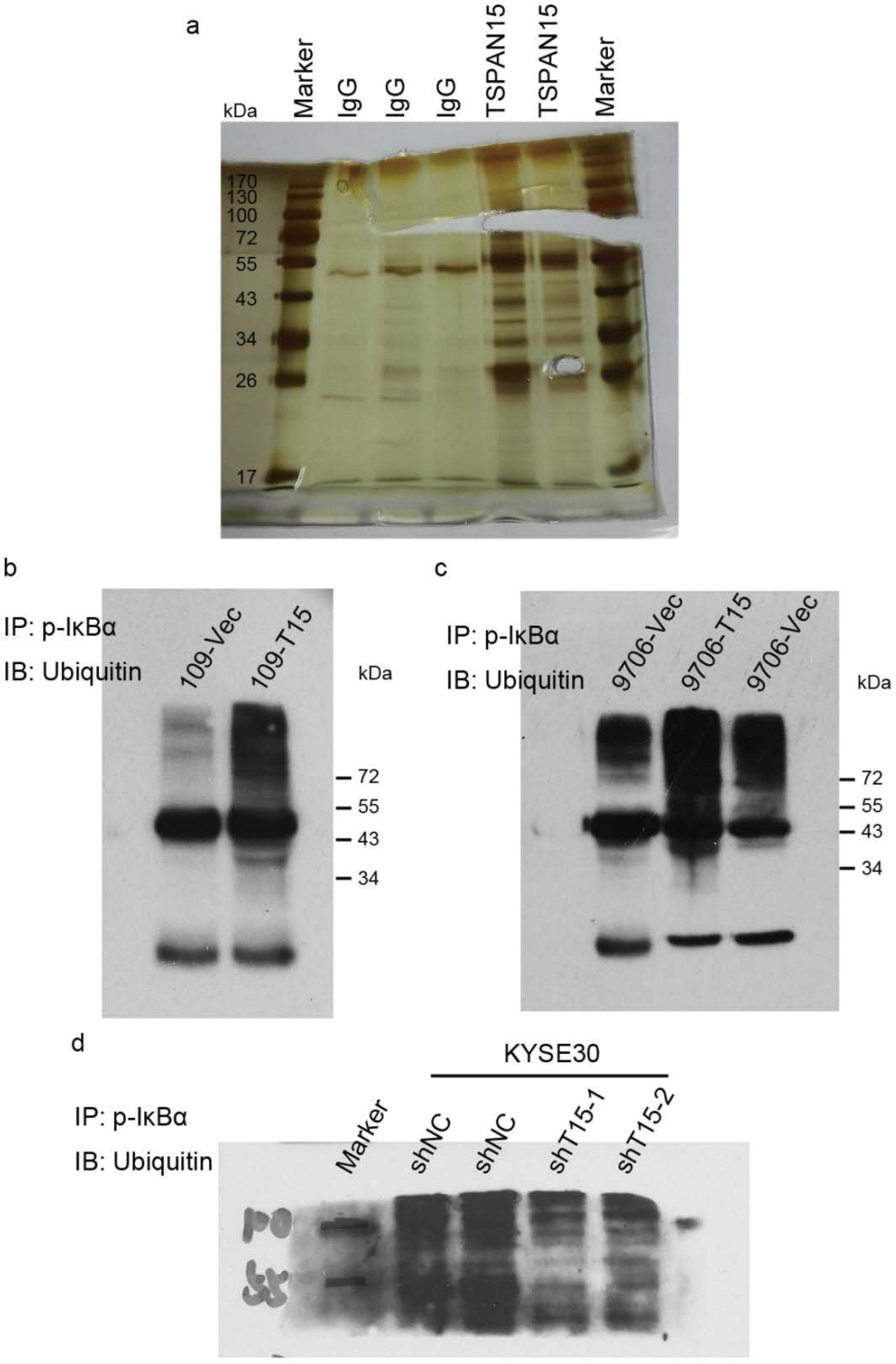

Supplementary Fig. 3. (a) Full gel of Fig. 3a. **(b-d)** Full blots of Fig. 3e.

Supplementary Table 1. List of antibodies used in this project

Antibody	Cat. No.	Vendor	Application
Rabbit anti-human TSPAN15	PAB24351	Abnova	WB, 1:1000; IHC, 1:100
Rabbit anti-human β-TrCP	#4394	Cell signaling	WB, 1:1000; IF, 1:100
Mouse anti-human IKKα	#11930	Cell signaling	WB, 1:1000
Rabbit anti-human IKKβ	#8943	Cell signaling	WB, 1:1000
Rabbit anti-human Phospho-IKKα/β (Ser176/180)	#2697	Cell signaling	WB, 1:1000
Rabbit anti-human NF-κB p65	#8242	Cell signaling	IF, 1:400
Mouse anti-human IκBα	#4814	Cell signaling	WB, 1:1000
Rabbit anti-human Phospho-IκBα (Ser32)	#2859	Cell signaling	WB, 1:1000
Mouse anti-human Ubiquitin	#3936	Cell signaling	WB, 1:1000
Mouse anti-Flag	F2555	MilliporeSigma	IF, 1:400
Mouse anti-human ICAM1	60299-1-Ig	Proteintech	WB, 1:1000; IHC, 1:50
Rabbit anti-human VCAM1	Ab106777	abcam	WB, 1:100
Rabbit anti-human VCAM1	11444-1-AP	Proteintech	IHC, 1:200
Rabbit anti-human uPA	ab169754	abcam	WB, 1:1000
Rabbit anti-human MMP9	AP6214a	ABGENT	WB, 1:1000; IHC, 1:200
Rabbit anti-human TNFα	ab9635	abcam	WB, 1:1000; IHC, 1:200
Rabbit anti-human MCP1	ab9669	abcam	WB, 1:1000; IHC, 1:200
Rabbit anti-human β-actin	#4970	Cell signaling	WB, 1:1000
Goat anti-mouse IgG HRP	#7074	Cell signaling	WB, 1:3000
Goat anti-rabbit IgG HRP	#7076	Cell signaling	WB, 1:3000
AF™ 488 donkey anti-mouse IgG	A-21203	Thermo Fisher Scientific	IF: 1:100
AF™ 488 goat anti-rabbit IgG	A-11008	Thermo Fisher Scientific	IF: 1:100
